# Kaliziri extract upregulates tyrosinase, TRP-1, TRP-2 and MITF expression in murine B16 melanoma cells

**DOI:** 10.1186/1472-6882-14-166

**Published:** 2014-05-22

**Authors:** Adila Tuerxuntayi, Yong-qiang Liu, Ablajan Tulake, Maidina Kabas, Aiden Eblimit, Haji Akber Aisa

**Affiliations:** 1 The Key Laboratory of Plant Resources and Chemistry of Arid Zone, Xinjiang Technical Institute of Physics and Chemistry, Chinese Academy of Sciences, Urumqi 830011, China; 2 State Key Laboratory Basis of Xinjiang Indigenous Medicinal Plants Resource Utilization, Urumqi 830011, China; 3 Human Genome Sequencing Center Department of Molecular and Human Genetics Baylor College of Medicine, Houston, TX 77030, USA; 4 University of the Chinese Academy of Sciences, Beijing 100039, China

**Keywords:** KZE, Melanogenesis, B16 melanoma cells, TYR, MITF

## Abstract

**Background:**

Kaliziri extract (KZE) is a traditional Uyghur medicine (TUM), used by traditional hospitals in China as an injection for treatment of vitiligo for more than 30 years. Clinical application has shown that this medicine has obvious therapeutic effects. However, its phytochemical analysis and mechanism have not been examined.

**Methods:**

KZE was extracted from seeds of Kaliziri [*Vernonia anthelmintica* (L.) Willd.] in ethanol-water (80:20, v/v), its components were identified by LC-MS/MS, and the signaling pathway of melanin synthesis in KZE treated murine B16 melanoma cells was examined by western blotting.

**Results:**

Liquid chromatography-mass spectrometry analysis confirmed that the main components of KZE are flavonoids. KZE increased the tyrosinase activity and melanin content in a dose-dependent manner at concentrations of 5-40 μg/ml, and treatment with 20 μg/ml of KZE enhanced the expression of tyrosinase in B16 cells in a time-dependent manner.

**Conclusions:**

KZE induced melanogenesis by increasing the expression of TYR, TRP-1, TRP-2 and MITF in B16 cells.

## Background

Traditional Uyghur medicine (TUM), one of the main medicinal systems in central Asia, is based on four humors: fire, air, water and earth, which generate four different body fluids: blood, phlegm, yellow bile and black bile [1]. The main ingredients of TUM are flowers, seeds, fruits, minerals and animal compartments. According to the TUM theory, diseases or impairments result from an imbalance between the four body fluids. TUM herbal formulation can regulate the balance of body fluids and cure diseases [2].

Kaliziri [*Vernonia anthelmintica* (L.) Willd.] is a plant that only grows in high-altitude areas of southern Xinjiang and small regions in Pakistan and India. Kaliziri is an erect, pubescent, annual herb, and its length is up to 90 cm. The leaves of this plant are elliptic lanceolate, 5 to 9 cm long and 2.5 to 3.2 cm wide, with apex acute, base tapering into the petiole, margins coarsely serrated, and pubescence found on both surfaces of the leaf. The Kaliziri florets are hairy and are violet or purple in color, and blossom in abundance, with homogenous, solitary, axillary or terminal heads that are 1.3 to 2 cm in diameter, with a linear bract near the top of the peduncle. The fruit are 4.5 to 6 mm long, oblong-cylindrical in shape and have 10 pubescent ribs.

Kaliziri is a Uyghur medicinal plant vastly used for treating diseases and is considered as highly therapeutic. The seeds are used as febrifuge for treating skin diseases like leukoderma (also named vitiligo) in traditional therapy. Vitiligo is called “baras” or “akbaras” in TUM. TUM has a long history of several centuries; its origin can be traced back to antiquity. During a long course of struggling with diseases, TUM evolved into a unique and integrated theoretical system. However, TUM also has a unique method and theory in the treatment of vitiligo. As recorded in the Uyghur medicine classics, “Maksini-adiwiya” (Persian, Muhammad huseyin. Mu calendar 1183, AD 1763), the ancient Uyghur doctors accumulated a lot of experience with the Uyghur medical treatment of vitiligo using the traditional method and continuous innovation, and this experience has been widely implemented in the treatment of vitiligo at home and abroad because of its effectiveness. The TUM Kaliziri injection was extracted from Kaliziri seeds by a scientific method (Pharmacopoeia of the People’s Republic of China, Uyghur Medicine volume, product with code number approved by SFDA:Z20063652) [3]. Its main components are flavonoids. Clinical application over many years has shown that this medicine has a significant therapeutic effect on vitiligo; hence Kaliziri extract is a commonly used drug for the treatment of vitiligo. This drug has been used for more than 30 years in Xinjiang. It is safe and reliable, as unlike drugs such as the psoralen class, it has no side effects on important organs such as liver and kidney, and children can use it. According to the TUM theory, Kaliziri can treat diseases through balancing the Mizaj in the liver, and eliminating abnormal Balgham Hilit (damp and cold) by excreting dampness. It is capable of regulating abnormal balgham by promoting blood circulation and coloring by increasing melanin cell function. Nonetheless, its effect and underlying mechanisms in melanogenesis are not very clear. The aim of this study was to clarify the effect of Kaliziri extract (KZE) and its molecular mechanism in melanin biosynthesis in B16 melanoma cells.

Vitiligo is an acquired, progressive, multifactorial, depigmentation disorder characterized by the appearance of circumscribed white macules in the skin caused by chronic, progressive loss of functional melanocytes in the epidermis [4,5]. Vitiligo affects 1-2% of the population worldwide, with no predilection for gender or race, and usually starts in childhood or young adulthood. Manifestations begin before 20 years of age in 50% of the cases, while in 25% the onset is before 14 years of age [6].

The etiology of vitiligo is poorly understood. There appears to be a genetic predisposition in a non-Mendelian pattern, with a polygenic and multi factorial inheritance. Numerous factors have been implicated in the development of vitiligo, including: stress, trauma, exposure to sunlight, infections, malignancies, neural abnormalities, melatonin receptor dysfunction, impaired melanocyte migration, certain drugs, endocrine diseases and cytotoxic compounds. These causal factors may act independently or in concert [7]. Pigmentation of the skin serves a number of valuable functions; perhaps foremost among these is the photo protection of underlying tissues from ultraviolet (UV) radiation.

Melanocytes respond to a wide variety of intrinsic and extrinsic factors produced by the environment or by neighboring cells in the skin, including UV, melanocyte stimulating hormone (MSH), agouti signal protein (ASP), endothelin 1 (ET1), dickkopf 1 (DKK1), a wide variety of growth factors, and cytokines [8,9]. The essential function of tyrosinase in melanin biosynthesis has been known for many decades. Melanin biosynthesis is catalyzed by three melanocyte-specific enzymes: TYR, tyrosinase-related protein 1 (TRP-1) and TRP-2 [10,11]. TYR is the rate-limiting enzyme in melanogenesis [12], catalyzing the hydroxylation of tyrosine to form 3, 4-dihydroxyphenylalanine (DOPA), followed by oxidation of DOPA to produce DOPA-quinone [13]. Therefore, inhibitors of TYR have been used in cosmetics as skin-whitening agents [14]. TRP-2 acts as a dopachrome tautomerase and catalyzes the rearrangement of dopachrome to form 5, 6-dihydroxyindole-2-carboxylic acid (DHICA) [15], and TRP-1 oxidizes DHICA to produce carboxylate indole-quinon [16]. TRP-1 and TRP-2 also function in the biosynthesis of melanin downstream of TYR. The tyrosinase family genes, TYR, TRP-1 and TRP-2, are tightly regulated by microphthalmia-associated transcription factor (MITF) [11,17,18].

MITF is the most important transcription factor involved in the regulation of TYR gene expression, which is involved in the pigmentation, proliferation and survival of melanocytes [19,20], thus MITF plays a pivotal role in melanogenesis [21,22]. It has been reported to bind to the M-box within the TYR promoter, and thus up regulate TYR gene expression [23].

The current study identified the main components of KZE and investigated the effect of KZE on mushroom TYR activity. We also examined the TYR activity and melanin content in B16 melanoma cells, as well as the expression of TYR, TRP-1, TRP-2 and MITF in B16 cells.

## Methods

### Reagents

Dimethylsulfoxide (DMSO), mushroom tyrosinase, L-3, 4-dihydroxyphenylalanine (L-DOPA), and 3-(4, 5-dimethyl-thiazol-2-yl)-2, 5-diphenyl tetrazolium bromide (MTT) were purchased from SIGMA (St. Louis, MO, USA). β-actin antibodies were purchased from Cell Signaling Technology (Danvers, MA, USA) and horseradish peroxidase-conjugated secondary antibodies were from GE Healthcare (Piscataway, NJ, USA). TYR, MITF, TRP-1 and TRP-2 antibodies were from Santa Cruz Biotechnology (Santa Cruz, CA, USA). Enhanced Bradford protein assay kit was from Beijing Biomed Co.LTD (Beijing, China). Phenylmethylsulfonyl fluoride (PMSF) and the components of the whole cell lysis buffer for western blot analysis were purchased from SIGMA (St. Louis, MO, USA).

### Preparation of KZE

The whole plants of Kaliziri were identified by professor Guanmian Sheng, Xinjiang Institute of Ecology and Geography, Chinese Academy of Science, China. A voucher specimen of the sample (No. VAW100920) is kept in the Xinjiang Key Laboratory of Plant Resources and Natural Products Chemistry, Xinjiang Technical Institute of Physics and Chemistry, Chinese Academy of Sciences, China.

250 g of Kaliziri [*Vernonia anthelmintica* (L.) Willd.] seeds (identify by Prof. Guan-mian Sheng from Xinjiang Institute of Ecology and Geography, Chinese Academy of Sciences) were soaked in 2.5 L of ethanol–water (80:20, v/v) for 1h at room temperature. Then, the extract was filtered and the filtrate was evaporated under reduced pressure using centrifuge to obtain the ethanol extract, with a yield of 3.38% w/w of the dry weight of the seeds (Pharmacopoeia of the People’s Republic of China, Uyghur Medicine volume) [3].

After drying, a dark brown paste was obtained and then dissolved in PBS. A stock solution of KZE (5 mg/ml) was prepared in PBS for further applications.

### LC-MS/MS analysis

Liquid chromatography-mass spectrometry (LC-MS/MS) analysis was performed on an Agilent series 1100 HPLC instrument (Agilent, Waldbronn, Germany) coupled with a QSTAR Elite System (AB-Sciex, Framingham, MA, USA). The chromatographic separation was achieved on a Waters XSELECT C18 column (2.5 μm, ID 2.1 mm × 100 mm). Mobile phase A was water with 0.1% formic acid and mobile B was acetonitrile. The eluting conditions were optimized as follows: 0–3 min, 5% B; 3–53 min, 5–85% B; 53–63 min, 85–100%; 63–80 min, 100% B. The liquid flow rate was set at 0.2 ml/min. The HPLC effluent was introduced into a mass spectrometer without spitting. The column temperature was 25°C. The injection volume was 5 μl. A QSTAR Elite System Hybrid Quadrupole-TOF LC/MS/MS mass spectrometer coupled with electron spray ionization (ESI) interface was used to obtain the MS/MS data using Analyst QS 2.0 software. The ionization conditions were optimized and the following conditions were used: ion spray voltage (IS) 4500 V; curtain gas (CUR) 35 psi; collision gas (CAD) 5 psi; temperature (TEM) 450°C; ion source gas 1 (GS1) 60 psi; ion source gas 2 (GS2) 50 psi; declusterin potential (DP) 60 V; focusing potential (FP) 350 V; collision energy (CE) 60 V for positive ion mode. Ion spray voltage (IS)-4300 V; curtain gas (CUR) 35 psi; collision gas (CAD) 5 psi; temperature (TEM) 500°C; ion source gas 1 (GS1) 60 psi; ion source gas 2 (GS2) 50 psi; decluster in potential (DP)-60 V; focusing potential (FP)-350 V; collision energy (CE)-45 V for negative ion mode. The detection was conducted considering a mass range of 100–1500 m/z.

### Measurement of mushroom tyrosinase activity

The effects on mushroom tyrosinase activity were determined in a cell-free system using mushroom tyrosinase following the method reported by Aoki *et al.*[24]. Eighty microliters of mushroom tyrosinase at 25U/mol were used. After adding L-DOPA (2.5 mM), the reaction mixture was incubated for a further 20 min at 37°C. Tyrosinase activity was determined by the absorbance at 490 nm of the reaction mixture, and compared with the control value [24].

### Cell culture

The murine B16 melanoma cell line was obtained from CAS (Chinese Academy of Sciences, China). B16 cells were grown in DMEM medium (Gibco, Life Technologies, USA) supplemented with 10% heat-inactivated fetal bovine serum (Gibco), 100 U/ml penicillin and 100 μg/ml streptomycin (Hyclone, USA) in a humidified atmosphere with 5% CO_2_ at 37°C.

### Cell viability assay

Cell viability was determined using the MTT assay. B16 cells were plated in 96-well dishes at a density of 5 × 10^3^ cells per well. After 24 h, different concentrations of KZE were added and the cells were incubated for 48 h. Then, 10 μl of MTT (5 mg/ml in PBS) solution were added into each well and cells were incubated at 37°C for another 4 h. Following medium removal, 150 μl of DMSO were added to each well and plates were gently shaken for 10 min. Optical absorbance was determined at 570 nm with a Spectra Max M5 (Molecular Devices, USA). Absorbance of cells without treatment was regarded as 100% cell survival. Each treatment was performed in quintuplicate and each experiment was repeated three times.

### Tyrosinase activity and melanin content assay

Tyrosinase activity was estimated by measuring the rate of L-DOPA oxidation as previously reported [25]. B16 cells were seeded in a 12-well plate at a density of 2 × 10^5^ cells per well and allowed to attach for 24 h. Then, cells were treated with KZE for 48 h, washed with ice-cold PBS twice, trypsinized with 0.25% trypsin (Hyclone) and collected in an Ep tube. After centrifuged at 3,000 rpm for 5 min the cells were washed once with PBS, and then 200 μl of Tris-0.1% Triton X-100 (pH6.8) were added to each tube. All tubes were incubated at −20°C for 30 min, and then the lysates were centrifuged at 12,000 rpm for 15 min to obtain the supernatant for the tyrosinase activity assay. Protein concentrations were determined by the Bradford method with bovine serum albumin (BSA) as a standard. 100 ml of supernatant containing 10 μg total protein were added to each well in a 96-well plate, and then mixed with 100 μl of 0.1% L-DOPA in PBS (pH6.8). After incubation at 37°C for 1 h, the dopachrome was monitored by measuring the absorbance at 475 nm.

The total melanin in the cell pellet was dissolved in 100 ml of 1M NaOH/10% DMSO for 1 h at 80°C and solubilized melanin was measured at 470 nm.

### Western blot analysis

B16 melanoma cells were seeded in 60-mm dishes at a density of 1 × 10^6^ cells per dish and treated with 20 μg/ml of KZE for five periods of time (0,12,24,48 and 72 h). The dishes were washed twice with cold PBS and lysed in cold whole cell lysis buffer [1 mM phenylmethylsulfonyl fluoride (PMSF), 50 mM KCl, 1% NP-40, 25 mM HEPES (pH7.8) 100 μg/ml leupeptin, 20 μg/ml aprotinin, 125 μM DTT, 1 mM Na_3_VO_4_]. 30 μg of protein from each sample were added to sodium dodecyl sulfate (SDS) sample buffer and proteins were separated by 10% polyacrylamide gel electrophoresis. Following electrotransfer to polyvinylidene fluoride (PVDF) membranes, the membranes were blocked with 2% BSA and 0.1% Tween20 in 0.01 M Tris–HCl buffered saline (TBS) for 1 h at room temperature. After three washes with TBS containing 0.1% Tween20 (TBST), membranes were incubated overnight at 4°C with TYR, MITF, TRP-1, TRP-2 (diluted 1: 200) or β-actin (diluted 1:1000) antibodies in TBST containing 5% BSA. After three washes with TBST and three washes with TBS, the membranes were incubated with horseradish peroxidase-conjugated secondary antibodies at a dilution of 1:10000 for 1 h at room temperature. After washing with TBST and TBS, proteins were visualized by ECL western blotting detection reagents (GE Healthcare). Densitometric analysis was performed using Quantity One (Bio-Rad, Hercules, CA, USA) to scan the signals. Western blot assay results reported here are representative of at least three independent experiments.

### Statistical analysis

All data are expressed as mean ± SEM. Statistical analysis was performed with one-way ANOVA followed by Tukey’s *post hoc* test for multiple comparison tests. Significant differences were accepted when *P* < 0.05.

## Results and discussion

**Figure 1:**
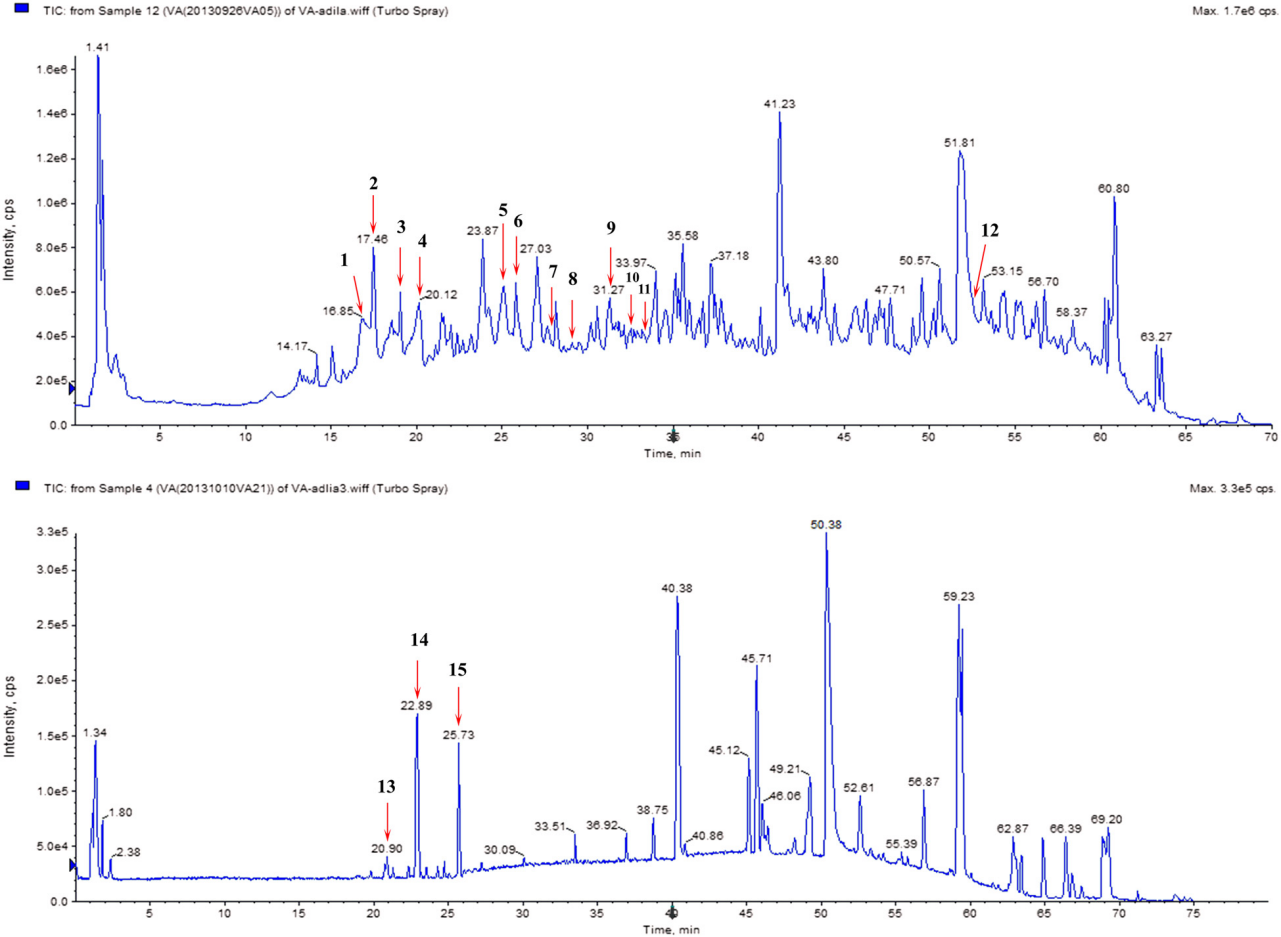
**LC–MS/MS analyze of KZE. Compounds detected in KZE in negative and positive MRM mode.** LC–MS/MS conditions as described in the text.

Kaliziri is a well-known herb, traditionally used as a pigmentation improving medicine for vitiligo in Xinjiang by the Uyghur people. KZE is extracted from Kaliziri seeds, and here we show that its main components are flavonoids (Figure 1 and Table 1). Long-term clinical application has shown that this medicine has an obvious therapeutic effect on vitiligo. However, its mechanisms in melanogenesis remain unknown.

**Table 1:** LC–MS/MS characteristics of KZI in MRM mode

**No.**	**Compound**	**Peak retention time (min)**	**Mr (relative molar mass)**	**Precursor ion (*m/z*)**	**Product ion (*m/z*)**
1	4- *O*-caffeoylquinate	16.85	354	353.08	191.05
2	3-*O*-caffeoylquinate	17.46	354	353.06	191.04
3	5,7,3′,4′-tetrahydroxy-flavonone-3-*O*-glucoside	19.14	466	465.08	303.03
4	3′-methoxy-5,7,4′-trihydroxy-dihydrochalcone-3-*O*-rutinoside	20.13	628	627.41	465.19
5	3,4-di-*O*-caffeoylisoquinic acid	25.06	516	515.05	353.06
6	3,4-di-*O*-caffeoylquinic acid	25.81	516	515.05	353.09
7	Liquiritigenin	27.93	256	255.06	119.04
8	Luteolin	29.19	286	285.05	150.99
9	Butein	31.29	272	271.05	135.04
10	Apigenin	32.44	270	269.06	135.01
11	Methoxyisorhamnetin	33.51	330	329.23	199.12
12	Kaempferide	52.71	300	299.24	165.00
13	Vernodalinol	20.91	378	401.14	277.12
14	Vernodalol	22.89	392	415.11	291.10
15	Vernodalin	25.73	360	361.17	259.10

### Liquid chromatography-mass spectrometry analysis of KZE

In our study, 15 compounds composing KZE were identified and characterized by LC-MS/MS (Figure 1 and Table 1). Eight of them are flavonoids: 5, 7, 3′, 4′-tetrahydroxy-flavonone-3-O-glucoside, 3′-methoxy-5,7,4′-trihydroxy-dihydrochalcone-3-O-rutinoside, liquiritigenin, luteolin, butein, apigenin, methoxy isorhamnetin, kaempferide. The analysis confirmed that the main components of KZE are flavonoids. In the next phase of the study, the sources and structures of these compounds need to be confirmed, to find out which are the active materials.

### Effects on mushroom tyrosinase activity

**Figure 2:**
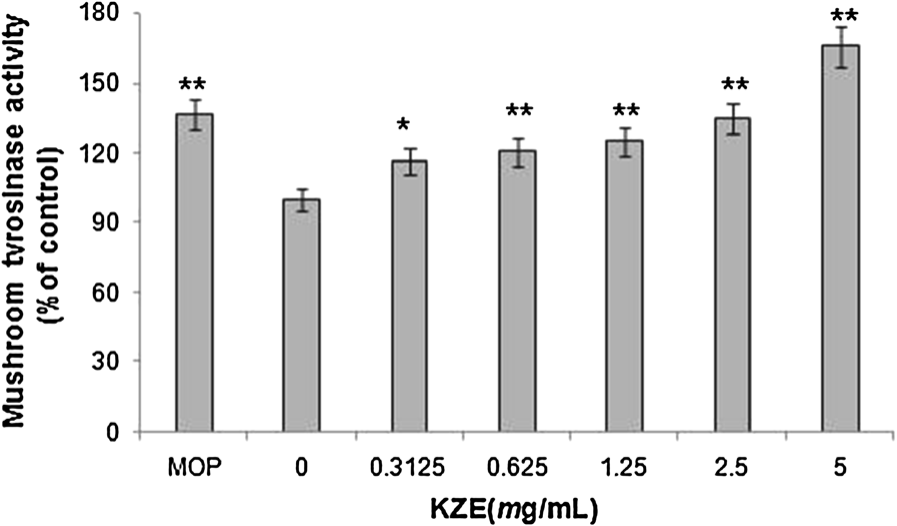
**The effect of KZE on mushroom tyrosinase activity.** Mushroom tyrosinase activity was determined by L-DOPA oxidation in a cell-free system. Stimulation of tyrosinase activity in vitro by KZE at 0.3125-5 mg/ml. MOP being positive controls at 500 *μ*M. Results shown are means ± SEM and are representative of three independent experiments. Data were analyzed by One-Way Analysis of Variance (ANOVA) followed by *post hoc* Tukey test. ^**^P < 0.01, compared with control.

The effect of KZE on mushroom tyrosinase activity was subsequently investigated *in vitro*. The results show that at five different concentrations KZE increased the tyrosinase activity in a dose-dependent manner (Figure 2).

### Cytotoxicity of KZE in B16 melanoma cells

**Figure 3:**
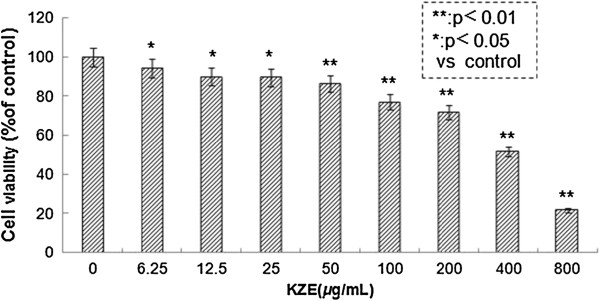
**Cytotoxicity of KZE in B16 melanoma cells.** Effect of KZE on B16 cell viability. B16 cells were treated for 48 h with various concentrations of KZE (6.25-800 μg/ml) and cell viability was determined by the MTT reduction assay. Data are expressed as mean ± SD (n = 6).

The effect of KZE on the viability of B16 melanoma cells was examined using the MTT assay. The cells were treated with various concentrations of KZE (6.25, 12.5, 25, 50, 100, 200, 400 and 800 μg/ml). As shown in Figure 3, the IC_50_ of KZE is 413 μg/ml, and there was no significant difference between the control and treated group at concentrations of 6.25–200 μg/ml. KZE showed very small cytotoxic effects on B16 cells.

### Effect of KZE on tyrosinase activity and melanin synthesis in B16 cells

**Figure 4:**
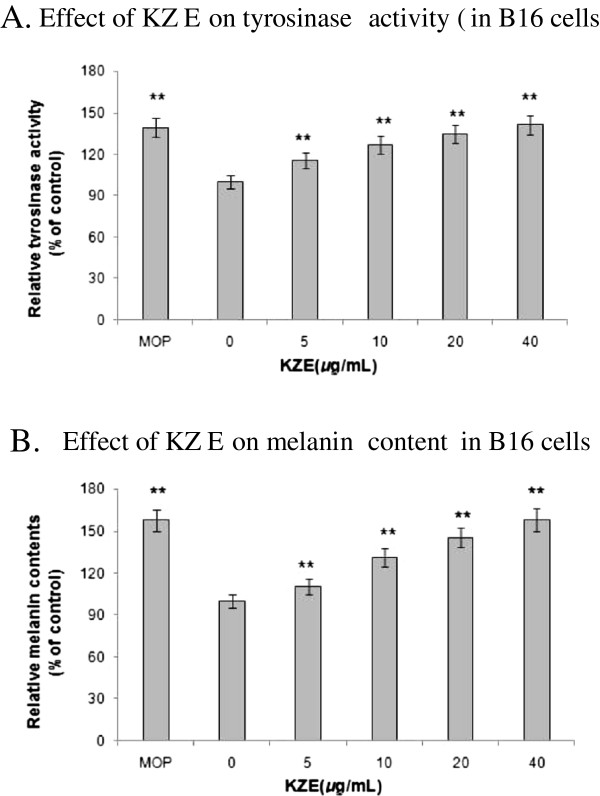
**Tyrosinase activity was determined by L-DOPA oxidation. A.** Stimulation of tyrosinase activity of B16 cell by KZE at 5-40 μg /ml. **B.** Melanin content were performed as described in "Materials and methods", B16 cells, the same by KZE at 5-40 μg /ml.MOP being positive controls at 50 μM. Results shown are means ± SEM and are representative of three independent experiments. Data were analyzed by One-Way Analysis of Variance (ANOVA) followed by post hoc Tukey test. **P<0.01, compared with control.

The effect of KZE on tyrosinase was measured by L-DOPA oxidation (Figure 3). Compared with treatment with medium only (untreated condition), treatment with KZE at 5–40 μg/ml resulted in a dose-dependent increase in tyrosinase activity in B16 cells (Figure 4A). In the melanin content assay, to exclude the possibility that a rise in melanin content maybe induced by the cell-proliferating effect of KZE, the absorbance of the same number of cells across KZE concentrations (5–40 μg/ml) was measured. We found that melanin levels increased in a dose-dependent manner by KZE treatment in B16 cells (Figure 4B). At 40 μg/ml of KZE, the melanin content only slightly increased, so 20 μg/ml was chosen as an effective concentration of KZE for further experiments.

### Effect of KZE on MITF and TYR protein expression in B16 cells

**Figure 5:**
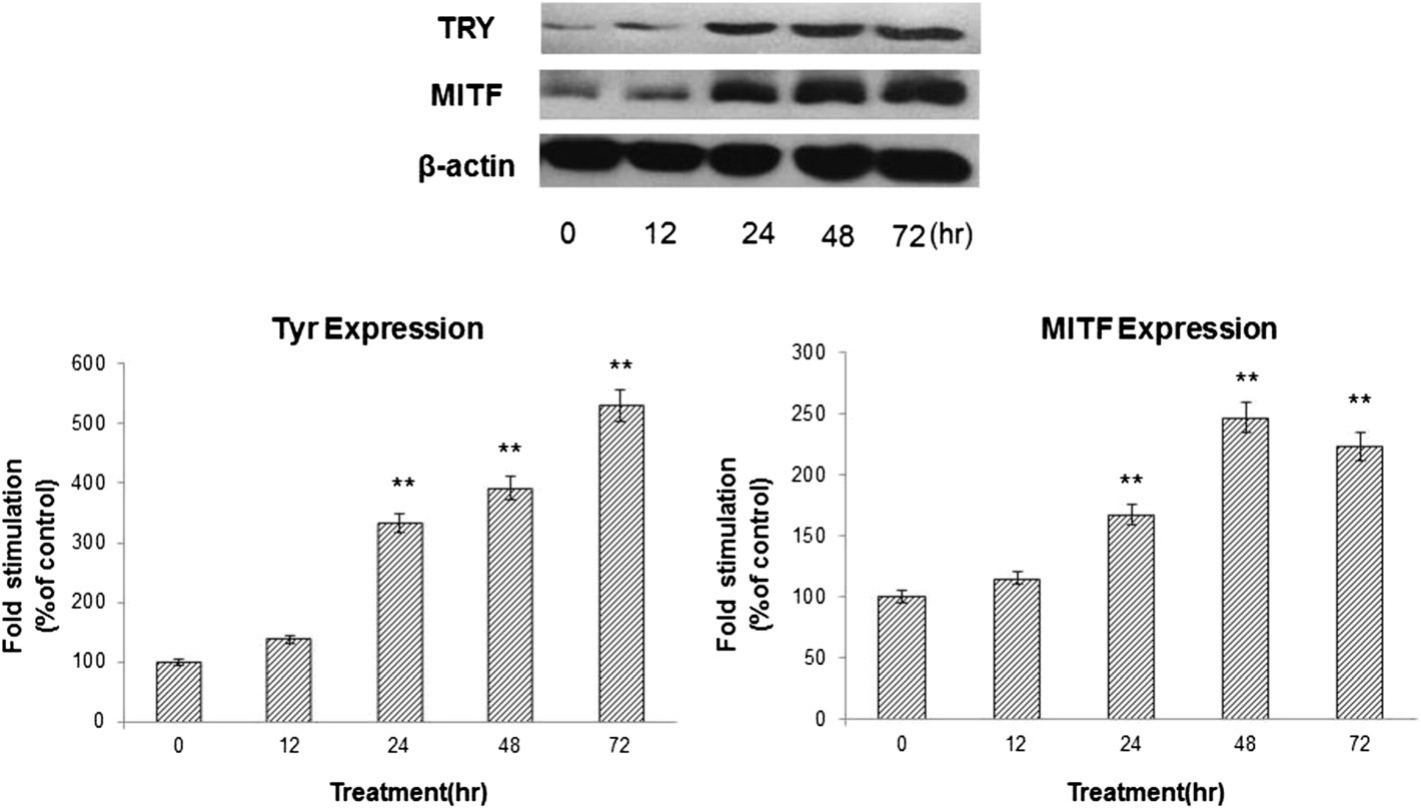
**Effect of KZE on the protein levels of MITF and tyrosinase in B16 cells.** The cells were treated with 20 μg /ml of KZE for the indicated times. Western blot assays were performed to examine MITF and tyrosinase expression levels. Results were normalized against β-actin expression. Results shown are means ± SEM and are representative of three independent experiments. Data were analyzed by One-Way Analysis of Variance (ANOVA) followed by *post hoc* Tukey test ^**^P < 0.01, compared with control.

Because KZE increased tyrosinase activity and melanin synthesis, we further explored whether KZE affects the expression of MITF, which plays a critical role in TYR gene expression and melanogenesis. We examined the MITF levels after KZE (20 μg/ml) treatment. Our data showed that MITF protein expression was significantly enhanced 24 h after KZE treatment of B16 cells (Figure 5). The effect of KZE on TYR expression in B16 cells was also examined by western blotting analysis. As shown in Figure 5, the level of TYR protein expression was up regulated by KZE treatment in a time-dependent manner.

### Effect of KZE on TRP-1 and TRP-2 protein expression in B16 cells

**Figure 6:**
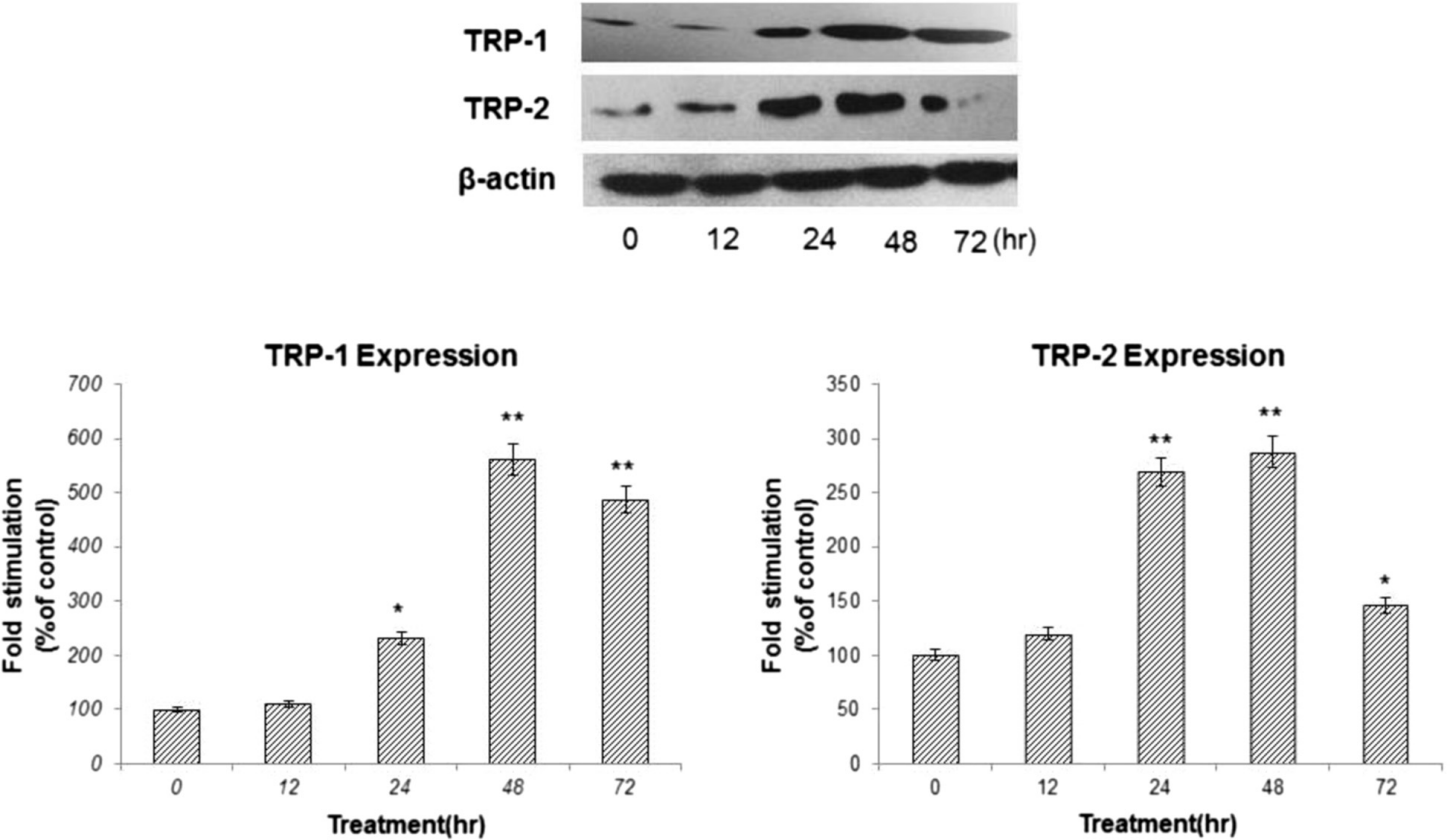
**Effect of KZE on the protein levels of TRP-1 and TRP-2 in B16 cells.** The cells were treated with 20 μg/ml of KZE for the indicated times. Western blot assays were performed to examine TRP-1 and TRP-2 expression levels. Results were normalized against β-actin expression. Results shown are means ± SEM and are representative of three independent experiments. Data were analyzed by One-Way Analysis of Variance (ANOVA) followed by *post hoc* Tukey test ^**^P < 0.01, compared with control.

To elucidate whether KZE can affect melanogenic protein expression, western blotting was carried out using lysates of B16 murine melanoma cells treated with KZE (20 μg/ml).The expression of TRP-1 and TRP-2 increased compared with the control (Figure 6). TRP-1 and TRP-2 protein expression was up regulated by KZE in a time-dependent manner in the B16 cell.

In our study, the positive effect of KZE on melanogenesis in B16 cell lines was investigated to clarify its underlying molecular mechanism. To evaluate the biological activity of KZE in melanin synthesis, we first examined its potential cytotoxicity in B16 cells. As shown in Figure 3, KZE had no cytotoxic effect at concentrations of 1–50 μg/ml, but it observably increased B16 cell proliferation. Because it has been reported that melanin content directly correlated with the activity of tyrosinase and its protein levels [26], the effect of KZE on tyrosinase activity and expression was further explored. As expected, KZE significantly increased both tyrosinase activity and melanin synthesis in a concentration-dependent manner (Figure 4A and B). These results suggest that KZE up regulated tyrosinase activity and enhanced cellular melanin synthesis in B16 cells. KZE affected tyrosinase activity at concentrations of 10–40 μg/ml in B16 cells (Figure 4A and B); we chose 20 μg/ml for the following experiments. To clearly elucidate the molecular mechanisms of KZE-induced actions, the effect of KZE on melanogenic protein expression was examined. As MITF plays an important role in melanogenesis as the major transcription regulator of TYR [27-29], the expression of MITF and TYR after treatment with 20 μg/ml KZE at 0–72 h was examined. As shown in Figure 5, KZE significantly increased TYR and MITF levels 72 h and 48 h after treatment, respectively.

TRP-1 and TRP-2 are transmembrane proteins spanning melanosomal membranes and may act together to modulate TYR activity. TRP-1 has been reported to influence TYR activity by forming a complex with it and/or stabilizing it [30]. TRP-2 functions as a dopachrome tautomerase downstream of TYR in the melanogenic pathway or the quantity and quality of the melanin produced during melanin biosynthesis [31]. As shown in Figure 6, KZE significantly increased the expression of TRP-1 and TRP-2 at 48 h in B16 cells.

## Conclusions

In conclusion, our results indicate that KZE induces melanogenesis by increasing the expression level of tyrosinase, TRP-1 and TRP-2 via MITF in B16 cells. The results provide an interesting insight into the mechanism of action of traditional Uyghur medicine in the treatment of vitiligo.

## Abbreviations

KZE: Kaliziri extract; TYR: Tyrosinase; TRP-1: Tyrosinase-related protein 1; TRP-2: Tyrosinase-related protein 2; MITF: Microphthalmia-associated transcription factor; EP tube: Eppendorf tube.

## Competing interests

The authors declare that they have no competing interests.

## Authors’ contributions

AT carried out the experimental work on the action mechanism of KZE and draft the manuscript. YL and AT carried out the LC-MS/MS analysis. HAA carried out the conceived of the study, and participated in its design and coordination and helped to draft the manuscript. EA helped to review the manuscript. All authors read and approved the final manuscript.

## Pre-publication history

The pre-publication history for this paper can be accessed here:

http://www.biomedcentral.com/1472-6882/14/166/prepub
